# Effects of Betaine Supplementation on Live Performance, Selected Blood Parameters, and Expression of Water Channel and Stress-Related mRNA Transcripts of Delayed Placement Broiler Chicks

**DOI:** 10.3389/fvets.2020.632101

**Published:** 2021-01-14

**Authors:** Ahmed Abdulaziz Al-Sagan, Abdulaziz Al-Abdullatif, Elsayed O. S. Hussein, Islam M. Saadeldin, Saud I. Al-Mufarrej, Mohammed Qaid, Hani H. Albaadani, Ayman Abdel-Aziz Swelum, Rashed Alhotan

**Affiliations:** ^1^King Abdulaziz City for Science and Technology, Riyadh, Saudi Arabia; ^2^Department of Animal Production, King Saud University, Riyadh, Saudi Arabia

**Keywords:** betaine, aquaporin, broiler chick, delayed placement, water channel

## Abstract

This study examined the effect of supplemental betaine on live performance, selected blood parameters, and gene expression of water channel proteins (Aquaporins, AQP) of broiler chicks delayed in placement for 48 h post-hatch. In total, 540 newly-hatched male broiler chicks were obtained from a local hatchery and were randomly allotted to one of five treatments with nine replicates per treatment (12 chicks per replicate). Chicks were either placed immediately, control; held for 48 h post-hatch with no access to feed or water, Holdnull; held for 48 h with free access to drinking water only, HoldW; held for 48 h with free access to drinking water supplemented with 1 ml per L of betaine solution (40% betaine), HoldB1; or held for 48 h with free access to drinking water supplemented with 2 ml per L of betaine solution (40% betaine), HoldB2 group. The results showed that post-hatch holding for 48 h depressed feed intake and body weight gain during the entire 15 d study period with no beneficial effect of supplemental betaine. Chicks in the HoldB2 group had elevated serum glucose, triglycerides, and aspartate aminotransferase 48 h post-hatch. Early water deprivation directly affected the brain proopiomelanocortin (POMC) and hepatic glucocorticoid receptors (GR) expression and induced significant changes in various aquaporins (AQP1, AQP2, AQP4, and AQP9). In conclusion, betaine supplementation to chicks held for 48 h post-hatch resulted in some changes in blood biochemical indices with no effects on performance during the first 15 days of life. The results suggest that betaine supplementation could ameliorate the stressful effects of water deprivation on POMC and GR expression and maintain cellular osmosis through interactions with variable aquaporins expression, particularly the AQP1 and AQP2. Further investigations are required to investigate the molecular mechanisms underlying the selective regulatory expression of different aquaporins in relation to betaine supplementation.

## Introduction

Under commercial settings, newly hatched broiler chicks experience periods of prolonged feed and water deprivation, which can last up to 72 h pre-placement. Besides the variable hatching window, other inevitable factors like sorting, vaccination, packaging, and transportation to rearing facilities contribute to the delayed placement. Delayed placement chicks have no access to feed in the hatchery or during transportation due to technical difficulties. However, water can be provided through various systems like Aqua Chick Tray (1). As a result, chick quality will deteriorate when chicks are exposed to dehydration, under-nutrition, and distress post-hatch. It has long been established that early access to feed and water is crucial for maximum growth performance and well-being of broiler chickens. Studies have indicated that chicks deprived of feed and water for 24 h or more post-hatch showed increased mortality rate (2), impaired immune system development (3), retarded growth (4, 5), and delayed gastrointestinal tract development (6).

Several attempts have been made to alleviate the detrimental effects of fasting by supplementing various products during post-hatch holding and transportation. Batal and Parsons (7) fed Oasis™ hatching supplement (70% moisture, 20% carbohydrate, and 10% CP) to chicks deprived of feed and water for 48 h post-hatch and observed an improvement in growth rate compared to chicks fasted for the same period. Henderson et al. (8) reported a 2.7% increase in final body weight of broilers fed hydrated EarlyBird™ supplement (14% moisture and 21% CP) for 24 h post-hatch compared to fasting chicks. Alhotan (9) did not observe any differences in growth performance at 22 days of age when a hatching supplement (76% moisture and 5% CP) was fed to broiler chicks as the sole source of nutrients compared to chicks fasted for 24 h post-hatch without any supplement. Other studies utilized various supplements to broiler chicks through water or feed with variable results (10–13). One important aspect of feeding hatching supplements to newly hatched chicks during holding and transportation is to prevent dehydration. Dehydration can be a real problem to chicks, mostly when shipped long distances. Chicks have a sizeable surface-area-to-volume ratio; therefore, they are more vulnerable to lose moisture faster when exposed to suboptimal environmental conditions (14). Therefore, newly hatched chicks should be kept hydrated at all times until water access is available.

Betaine or Trimethylglycine is a naturally occurring chemical compound found primarily in microbes and plants subjected to osmotic stress and drought to serve as an organic osmolyte. During high osmotic stress, water moves out of the cell toward the higher concentration of solutes (e.g., Na^+^) outside the cells (15, 16). Continuous loss of water will cause the cells to shrink in size and eventually die. Therefore, to preserve water and maintain the cells' integrity, the osmotic pressure has to be adjusted by osmolytes. Several reports have indicated that betaine supplementation to heat-stressed poultry exerted positive effects on biological performance (15, 17, 18). Supplementation of diets with betaine can improve feed intake, weight gain, feed conversion ratio and immunity of broiler reared under heat stress condition (19). However, the role of betaine in alleviating the adverse effects of post-hatch holding remains unclear and needs to be elucidated.

Water is the principal constituent of the chick body, representing about 76% of the body weight (BW) at hatch (20). Furthermore, water is required for many body processes like digestion, absorption, metabolism, and temperature regulation. For optimal functionality of such processes, water homeostasis and transport must be regulated. Water transport across biological membranes under osmotic gradient is carried out by selective water channels known as Aquaporins (21). The aquaporins are a group of porous integral membrane proteins found in almost all life forms and are composed of a bundle of six membrane-spanning α-helical domains with low molecular weights (22). In mammals, 13 isoforms of aquaporin have been discovered, and these are AQP0 to AQP12 (23). The presence of the aquaporins has been reported in various organs, including the kidney, brain, intestine, liver, and reproductive organs of poultry (24–27). Recently, Orlowski et al. (28) examined the expression of the aquaporin water channels in the kidney and blood in three lines of chickens subjected to 3 h of water restriction and found that water restriction altered the expression of aquaporin genes. Expression of aquaporin genes in newly hatched chicks subjected to delayed placement for 48 h and the role of betaine in aquaporin gene regulation is poorly investigated. Moreover, the role of betaine in improving the performance of the post-hatch held chicks is unclear. Therefore, the objective of this study was to investigate the effects of supplemental betaine during delayed placement of broiler chicks on growth performance, selected blood parameters, and the expression of selected water channel and stress-related mRNA transcripts.

## Materials and Methods

### Birds and Housing

All the procedures followed in this study were approved by the Research Ethics Committee at King Saud University (Riyadh, Saudi Arabia; Ethics Reference No: KSU-SE-20-54). A total of 540 newly-hatched Ross 308 male broiler chicks were obtained from a commercial hatchery. Chicks were housed in floor pens in three environmentally controlled rooms. Each pen measured about 1 m^2^ and was bedded with new pine wood shavings. All floor pens were equipped with a plastic hopper feeder and a bell drinker. The photoperiod provided was 24 h of light a day during the entire 15 d experiment. Room temperature was initially set at 30°C, and the temperature was gradually decreased to reach 24°C at the end of the experimental period. A common corn-SBM diet was formulated to meet the nutritional specifications of Ross (29); Aviagen and was fed in mash form (Table 1).

**Table 1:** Feed ingredients and calculated composition of the basal diet.

**Ingredient (%)**	**Starter (0–15 d)**
Yellow corn	56.32
Soybean meal (48%)	37.60
Vegetable oil	2.28
Dicalcium phosphate	1.62
Limestone	1.02
Dl-Methionine	0.31
L-Lysine HCL	0.15
L-Threonine	0.09
Common salt	0.40
Vitamin premix^1^	0.10
Mineral premix^2^	0.10
Calculated Composition	100.00
Metabolizable energy, kcal/kg	3050
Crude protein, %	22.25
Calcium, %	0.92
Available phosphorus, %	0.46
Digestible lysine, %	1.22
Digestible methionine, %	0.62
Digestible total sulfur AA, %	0.91
Digestible threonine, %	0.81
Digestible tryptophan, %	0.24
Digestible arginine, %	1.41
Digestible valine, %	0.95

1 *Vitamin premix provided the following (per kg of diet): Vitamin B1, 2 mg; Niacin, 50 mg; Vitamin B2, 6 mg; Pantothenic Acid, 15 mg; Vitamin B12,16.0 μg; Vitamin B6, 3 mg; Biotin, 150 μg; Folic Acid, 1.75 mg; Vitamin K3 (MNB), 3 mg; Vitamin D3 (cholecalciferol), 5000 IU; Vitamin A (retinol acetate), 10,000 IU; Vitamin E (Dl-alpha-tocopheryl acetate), 50 IU; Total antioxidants, 50 mg*.

2 *Trace mineral premix provides the following in milligrams per kilogram of diet: Manganese (Oxide), 120; Zinc (Oxide), 100; Iron (Sulfate), 40; Copper (Sulfate), 16; Iodine (Potassium Iodide), 1.25; Selenium (Sodium Selenite) 0.30*.

### Experimental Design and Treatment Design

The design of the experiment was a randomized complete block design with room (*n* = 3) as a blocking factor. There were five treatments: (1) Placing chicks immediately, control group; (2) Holding chicks for 48 h with no access to feed or water, Holdnull group; (3) Holding chicks for 48 h with free access to drinking water only, HoldW group; (4) Holding chicks for 48 h with free access to drinking water supplemented with 1 ml per L of a betaine solution, HoldB1 group; and (5) Holding chicks for 48 h with free access to drinking water supplemented with 2 ml per L of a betaine solution, HoldB2 group. Upon arrival, chicks were weighed and randomly assigned according to treatment to replicate pens. Chicks in Treatment 1 were placed immediately, whereas chicks in treatments 2 to 5 were held in shipping boxes with or without drinking water and then were placed after the holding time elapsed. Each treatment was replicated 9 times, with 12 chicks per replicate pen. Betaine was added to drinking water in liquid form as ActiBeet® L (AtcoBeet, ATCO PHARMA; Menoufia, Egypt) containing 40% natural betaine.

### Measurements and Sample Collection

BW of chicks and feed were weighed at 0, 5, 10, and 15 days of age. BW gain, feed intake, and feed conversion ratio were calculated accordingly. Mortality was collected and reported as it occurred. At 2 (after fasting for the fasted groups), and 15 days of age, one chick per replicate pen (9 per treatment) was randomly selected for blood and sample collection using sterilized needles and 1 ml syringes. Then, the blood samples were left to coagulate at room temperature and centrifuged at 3,500 rpm (2,328.24 × g) for 5 min to separate serum. The serum samples were then transferred into sterilized Eppendorf tubes and stored at −20°C for blood analysis. Tissue samples (≈1 g) from the euthanized chicks were taken from the brain, kidney, intestinal ileum (≈2 cm distal to Meckel's diverticulum), and liver. The samples were first rinsed in normal saline to remove any contaminants, placed into sterilized tubes filled with RNAlater solution (RNAlater, Ambion, Austin, TX), and then stored at −80°C for RNA extraction.

### Blood Biochemical Analysis

Serum biochemical concentrations were measured for total protein, albumin, uric acid, glucose, triglycerides, and aspartate aminotransferase. These biochemical parameters were analyzed by a spectrophotometric analyzer (Randox, London, UK) using reagent kits (Randox, London, UK) according to the manufacturer's instructions.

### RNA Isolation, cDNA Synthesis, and Real-Time Polymerase Chain Reaction

The total RNA was extracted from the tissue samples (brain, kidney, intestine, and liver) according to (30) using the PureLink™ RNA Mini Kit (Invitrogen, Carlsbad, CA, USA) following the manufacturer's protocol. The resulting RNA's concentration and purity were estimated using a Nanodrop 2000 spectrophotometer (Thermo Fisher, Waltham, MA, USA) to estimate the values at 230 and 260 nm, and the acceptable ratios were above 1.80. The total RNA was then reverse-transcribed into complementary DNA (**cDNA**) using the High-Capacity cDNA Reverse Transcription Kit (Applied Biosystems, Carlsbad, CA, USA) according to the manufacturer's instructions. Real-time PCR analysis was performed using the Power SYBR™ Green PCR Master Mix (Applied Biosystems), where β-actin was used as the internal control. Transcripts of chicken β-actin (**ACTB**), glucocorticoid receptors (**GR**), pro-opiomelanocortin (**POMC**), aquaporins (AQP1, AQP2, AQP3, AQP4, and AQP9) were amplified using gene-specific primer sequences (Table 2), and the qRT-PCR was performed using 20 μL reactions that contained 10 μL of 2X Power SYBR™ Green PCR Master Mix, 1 μL of each primer (10 pmol), 6 μL of water and a 2 μL cDNA template. This was performed with the following cycling conditions: 15 min at 95°C, followed by 40 cycles of 15 s at 95°C, 30 s at 60°C, and 30 s at 72°C. Melt-curve analysis was performed (from 65 to 95°C, using 0.5°C temperature increments with a 5 s hold at each step) using Applied Biosystem 7500 real-time PCR machine (Applied Biosystems). The relative fold-change in the expression of target genes was calculated using the comparative 2^−ΔΔCt^ method (31), where ΔΔCt indicates the difference between the mean ΔCt of the treatment group and that of the control group, and ΔCt represents the difference between the mean Ct of the gene of interest and that of the ACTB for each sample. Moreover, the melting curve showed a single peak for each primer pairs.

**Table 2:** The primers sequence and information used for real-time PCR analysis^1^.

**Gene name**	**Sequence 5^′^−3^′^**	**Size (bp)**	**Accession No**.
GR-F	TATGACAGCACGCTGCCCGA	76	NM_001037826
GR-R	CTACCACTTGCCGTCCTCCTAACAT		
ACTB-F	CCATCTATGAAGGCTACGC	124	NM_205518
ACTB-R	CTCGGCTGTGGTGGTGAA		
POMC-F	GGAAAAGAAGGATGGAGGCTC	155	NM_001031098
POMC-R	TCTTGTAGGCGCTTTTGACGAT		
AQP1-F	AGCTGGTTTTGTGCGTTCTT	143	NM_001039453.1
AQP1-R	TCTGGCTGGGTTAATTCCAC		
AQP2-F	TTTGCAGCCTCCATGATGTG	56	NM_001292072
AQP2-R	AGGACAGCCCGGGTGAA		
AQP3-F	TGCTCCTGGTCCCTGACACT	58	AB358970.1
AQP3-R	CTTTTGCCTTCCCATTGCA		
AQP4-F	CGCTCGCAGCAGCAGTAA	59	NM_001317827
AQP4-R	ATGCTACCATGATGCTCTCACACT		
AQP9-F	CACAATTCCTGGGAGCATTT	110	AB359226.1
AQP9-R	TGTTGCGTAAGGTCCTGTGA		

1 *F, forward primer; R, reverse primer; bp, base pair; ACTB, beta actin; GR, glucocorticoids receptor; POMC, proopiomelanocortin; AQP, aquaporin*.

### Statistical Analysis

Data were analyzed using the GLM procedure of SAS software 9.2 (32). The one-way analysis of variance was used for hypothesis testing, and the MEANS Procedure was employed to compute the descriptive statistics of the response variables. Mean separation was performed with Tukey's Studentized Range (HSD) Test. Data for gene expression was plotted using SigmaPlot 14.0 (33). Significant differences were considered when *P* ≤ 0.05. Pearson's linear correlation coefficients were calculated to determine the correlation (r) between the means of different mRNA transcript expressions, with *r* values > ± 0.7, strong positive/negative linear relationship; *r* > ± 0.5 and ≤ ±0.7, moderate positive/negative linear relationship; or *r* < ±0.5, weak positive/negative linear relationship (34). Percentages mortality rates were transformed using Arcsine transformation before analysis.

## Results

### Growth Performance

Growth performance results are presented in Table 3. Chicks that were delayed in placement (treatment groups 2 to 5) consumed less (*P* < 0.001) feed during the entire 15-day experiment compared to the early placed chicks of the control group. Similarly, the delayed chicks gained less (*P* < 0.001) weight at all points of time examined compared to the control group with no differences between the delayed groups. Feed conversion ratio did not differ (*P* > 0.05) between treatment groups during the periods 0–5 and 5–10 d of age. However, chicks delayed in placement and supplemented with 2 ml of betaine (HoldB2 group) had reduced feed conversion ratio compared to the control group during the last period examined (10-15 d; *P* = 0.026) as well as during the whole period (0-15 d; *P* = 0.020). There were no significant differences between treatment groups in terms of mortality rates.

**Table 3:** Effect of betaine supplementation in drinking water on the live performance of broiler chicks held for 48 h post-hatch.

**Variable**	**Treatments^1^^,^^2^^,^^3^**					***P*-value**	**SEM**
	**Control**	**Holdnull**	**HoldW**	**HoldB1**	**HoldB2**		
**0 to 5**							
Feed intake (g/chick)	81.6^a^	37.8^b^	38.0^b^	40.0^b^	37.9^b^	<0.001	1.3
Body weight gain (g/chick)	67.9^a^	29.2^b^	32.3^b^	31.3^b^	31.9^b^	<0.001	1.3
Feed conversion ratio (g/g)	1.21	1.30	1.18	1.29	1.19	0.148	0.04
**5 to 10**							
Feed intake (g/chick)	191.1^a^	139.3^b^	144.6^b^	140.2^b^	141.2^b^	<0.001	2.7
Body weight gain (g/chick)	163.2^a^	120.3^b^	125.1^b^	124.8^b^	123.1^b^	<0.001	2.8
Feed conversion ratio (g/g)	1.17	1.16	1.16	1.13	1.15	0.355	0.02
**10 to 15**							
Feed intake (g/chick)	333.4^a^	268.7^b^	278.5^b^	269.8^b^	271.7^b^	<0.001	4.2
Body weight gain (g/chick)	283.3^a^	235.4^b^	247.1^b^	238.8^b^	242.3^b^	<0.001	4.6
Feed conversion ratio (g/g)	1.18^a^	1.14^ab^	1.13^ab^	1.13^ab^	1.12^b^	0.026	0.01
**0 to 15**							
Feed intake (g/chick)	606.1^a^	445.8^b^	461.1^b^	450.0^b^	450.7^b^	<0.001	6.5
Body weight gain (g/chick)	514.4^a^	384.9^b^	404.5^b^	395.0^b^	397.3^b^	<0.001	6.7
Feed conversion ratio (g/g)	1.18^a^	1.16^ab^	1.14^ab^	1.14^ab^	1.13^b^	0.020	0.01
Mortality rate (%)	2.22	1.11	2.22	3.33	3.33	0.863	1.83

1 *Control, Placed on feed immediately; Holdnull, Holding for 48 h + No access to feed or water; HoldW, Holding for 48 h + water only; HoldB1, Holding for 48 h + water supplemented with betaine (1 ml per L); HoldB2, Holding for 48 h + water supplemented with betaine (2 ml per L)*.

2 *Values are means of 8 replicate pens per treatment*.

3 *Means within a row with no common superscript differ significantly (P ≤ 0.05)*.

### Blood Biochemical Parameters

At 48 h post-hatch, chicks delayed in placement and supplemented with 2x betaine (HoldB2 group) had lower (*P* < 0.001) serum total protein levels compared to their fed counterparts of the control group and had the lowest total protein among the delayed groups (Table 4). Chicks deprived of both feed and water (Holdnull group) were found to have elevated albumin levels and were higher (*P* < 0.001) than those of the HoldW and HoldB2 groups. Serum uric acid levels were higher (*P* < 0.001) in the Holdnull and HoldB2 groups than the control with the other groups being intermediate. The HoldB2 group had remarkably higher (*P* = 0.007) blood glucose concentration than the Holdnull group. Interestingly, delayed placement resulted in an elevation in the serum triglycerides with betaine supplementation having more pronounced effects (*P* < 0.001) on triglycerides levels. Moreover, aspartate aminotransferase tended to increase with feed deprivation and was higher (*P* = 0.021) in the HoldB2 group than the control. At day 15 post-hatch, treatment effects on most of the selected blood parameters vanished. However, uric acid was found to be increased (*P* = 0.008) in the HoldB1 group compared to the others. Furthermore, triglycerides for the HoldW, HoldB1, and HoldB2 groups were still higher (*P* < 0.001) compared to the control.

**Table 4:** Effect of betaine supplementation in drinking water on selected blood biochemical parameters of broiler chicks held 48 h post-hatch.

**Variable**	**Treatments^1^^,^^2^^,^^3^**					***P*-value**	**SEM**
	**Control**	**Holdnull**	**HoldW**	**HoldB1**	**HoldB2**		
**Day 2**							
Total protein (g/dl)	3.51^ab^	3.64^a^	3.28^cb^	3.58^a^	3.12^c^	<0.001	0.07
Albumin (g/dl)	1.44^ab^	1.66^a^	1.25^b^	1.41^ab^	1.22^b^	<0.001	0.06
Uric acid (mg/dl)	4.46^c^	8.05^a^	5.75^bc^	5.83^bc^	6.47^b^	<0.001	0.38
Glucose (mg/dl)	225.78^ab^	201.48^b^	227.76^ab^	226.59^ab^	256.79^a^	0.007	9.65
Triglycerides (mg/dl)	54.46^d^	57.00^d^	84.43^c^	99.00^b^	137.34^a^	<0.001	3.52
Aspartate aminotransferase (U/L)	219.87^b^	243.07^ab^	240.60^ab^	257.67^ab^	275.87^a^	0.021	11.53
**Day 15**							
Total protein (g/dl)	2.51	2.48	2.45	2.58	2.48	0.796	0.08
Albumin (g/dl)	1.36	1.32	1.08	1.26	1.28	0.144	0.08
Uric acid (mg/dl)	3.00^b^	3.53^ab^	3.23^b^	4.54^a^	3.22^b^	0.008	0.30
Glucose (mg/dl)	255.98	261.57	270.90	264.07	255.68	0.690	8.41
Triglycerides (mg/dl)	64.14^c^	84.47^c^	113.167^b^	133.49^ab^	152.50^a^	<0.001	5.27
Aspartate aminotransferase (U/L)	197.36	197.09	202.01	212.81	210.24	0.371	6.95

1 *Control, Placed on feed immediately; Holdnull, Holding for 48 h + No access to feed or water; HoldW, Holding for 48 h + water only; HoldB1, Holding for 48 h + water supplemented with betaine (1ml per L); HoldB2, Holding for 48 h + water supplemented with betaine (2 ml per L)*.

2 *Values are means of 9 chicks per treatment*.

3 *Means within a row with no common superscript differ significantly (P ≤ 0.05)*.

### Expression of Water Channel and Stress-Related Transcripts

**Figure 1:**
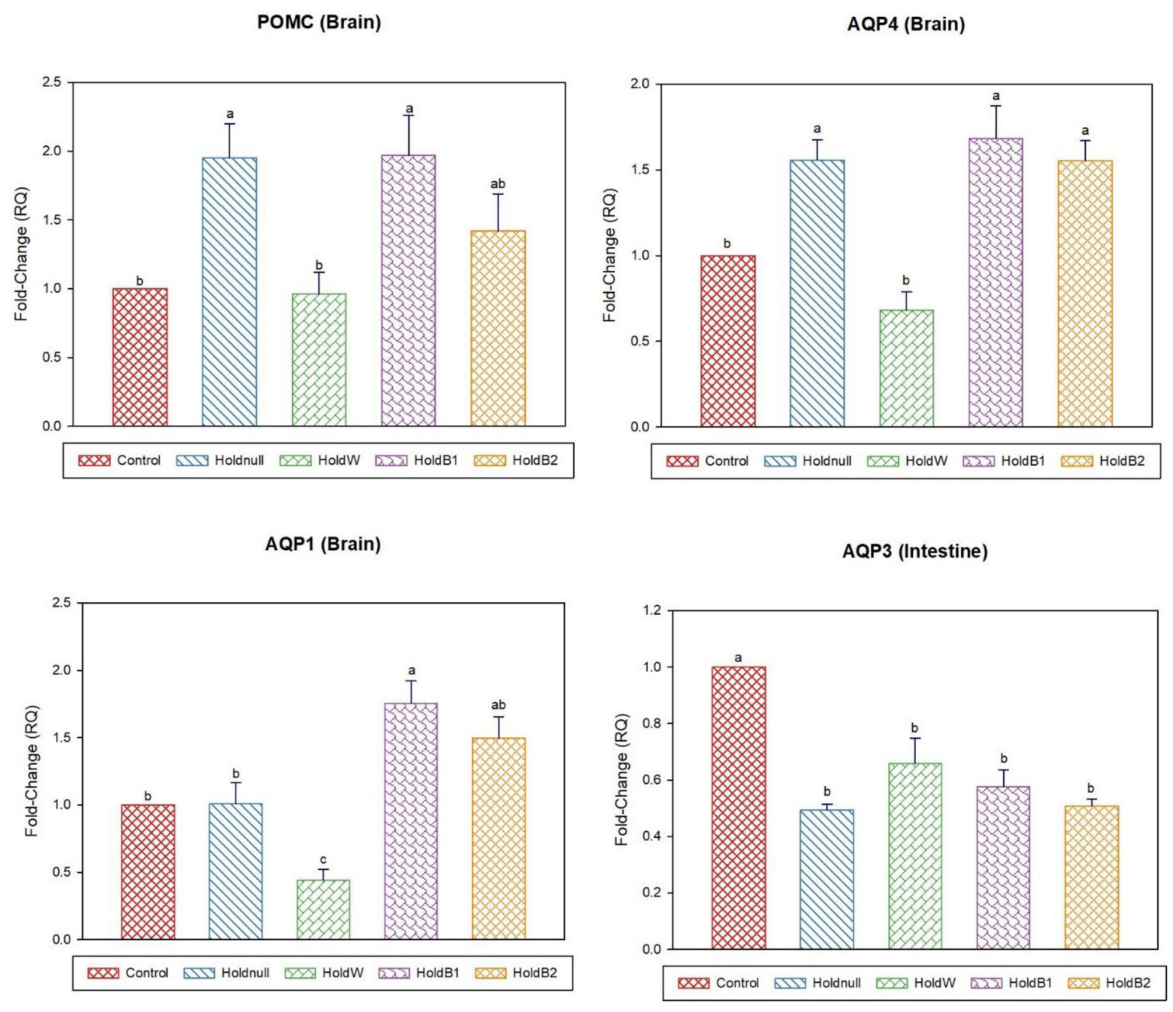
Effect of betaine on the relative expression (fold-change or RQ) of POMC, AQP4, AQP1 in the brain, and AQP3 in the intestine of day-2 old chicks. Control, placed on feed immediately; Holdnull, holding for 48 h + no access to feed or water; HoldW, holding for 48 h + water only; HoldB1, holding for 48 h + water supplemented with betaine (1 ml per L); HoldB2, holding for 48 h + water supplemented with betaine (2 ml per L). Bars with different letters are significantly different (*p* < 0.05). Mean ± standard error of the mean, *n* = 6. AQP, aquaporin; POMC, proopiomelanocortin.

**Figure 2:**
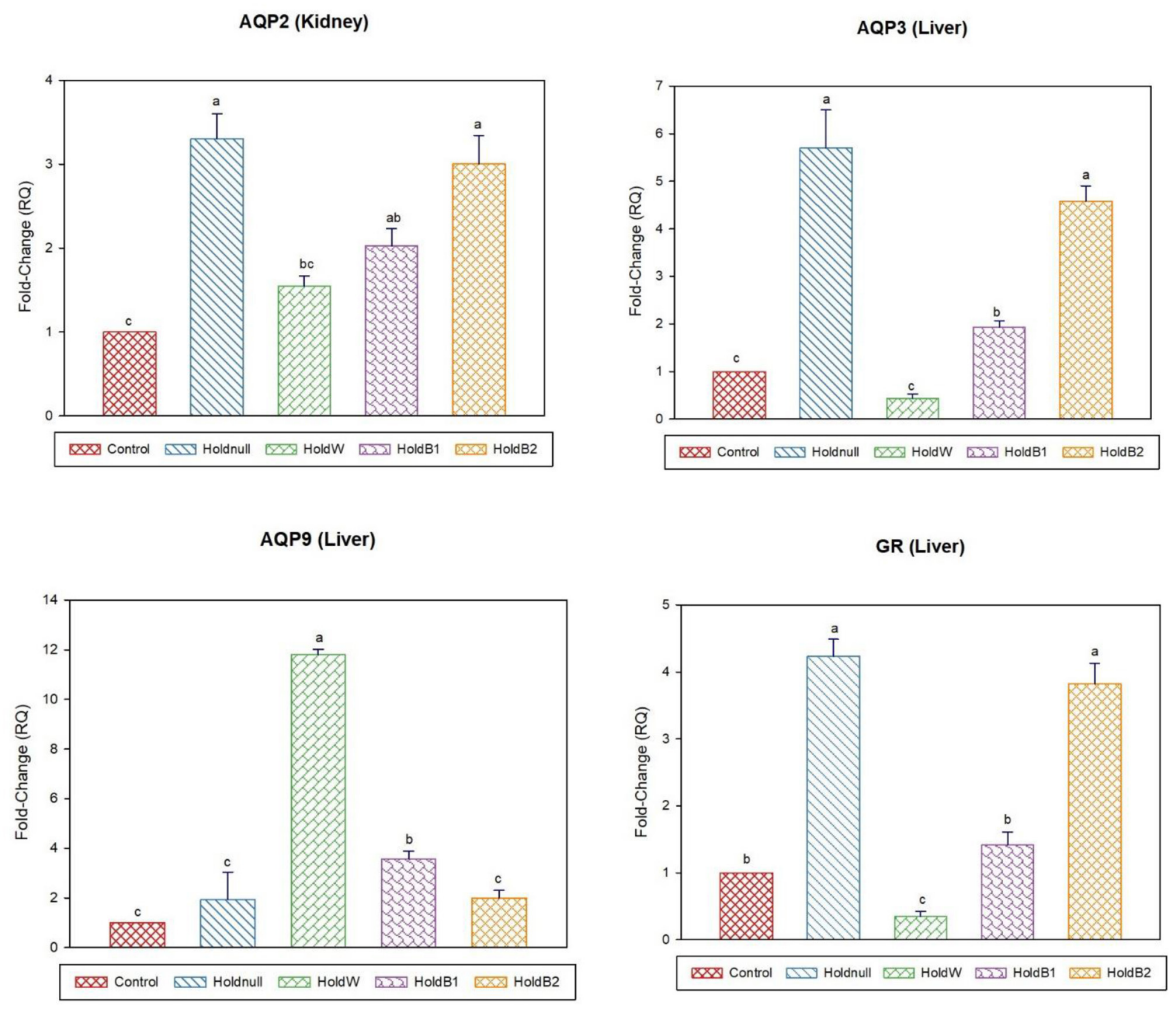
Effect of betaine on the relative expression (fold-change or RQ) of *AQP3, AQP9, GR* in the liver, and *AQP2* in the kidney of day-2 old chicks. Control, placed on feed immediately; Holdnull, holding for 48 h + no access to feed or water; HoldW, holding for 48 h + water only; HoldB1, holding for 48 h + water supplemented with betaine (1 ml per L); HoldB2, holding for 48 h + water supplemented with betaine (2 ml per L). Bars with different letters are significantly different (*p* < 0.05). Mean ± standard error of the mean, *n* = 6. AQP, aquaporin; GR, glucocorticoids receptor.

On day-2, water holding increased the expression of brain AQP4, renal AQP2, and hepatic AQP3, while there was no effect on brain AQP1 and hepatic AQP9 (Figures 1, 2). Conversely, water holding reduced intestinal AQP3. Moreover, water holding increased both POMC in the brain and the GR in the liver. Water supplementation ameliorated the impacts of water holding on the expression of brain POMC and AQP4, renal AQP2, and hepatic AQP3 mRNA transcripts with a significant reduction in brain AQP1 and hepatic GR. At the same time, hepatic AQP9 showed a significant increase. Betaine-supplemented water during holding partially maintained the effects of water holding on aquaporins with a prominent relief on the effects on brain POMC with increased betaine dosage.

**Figure 3:**
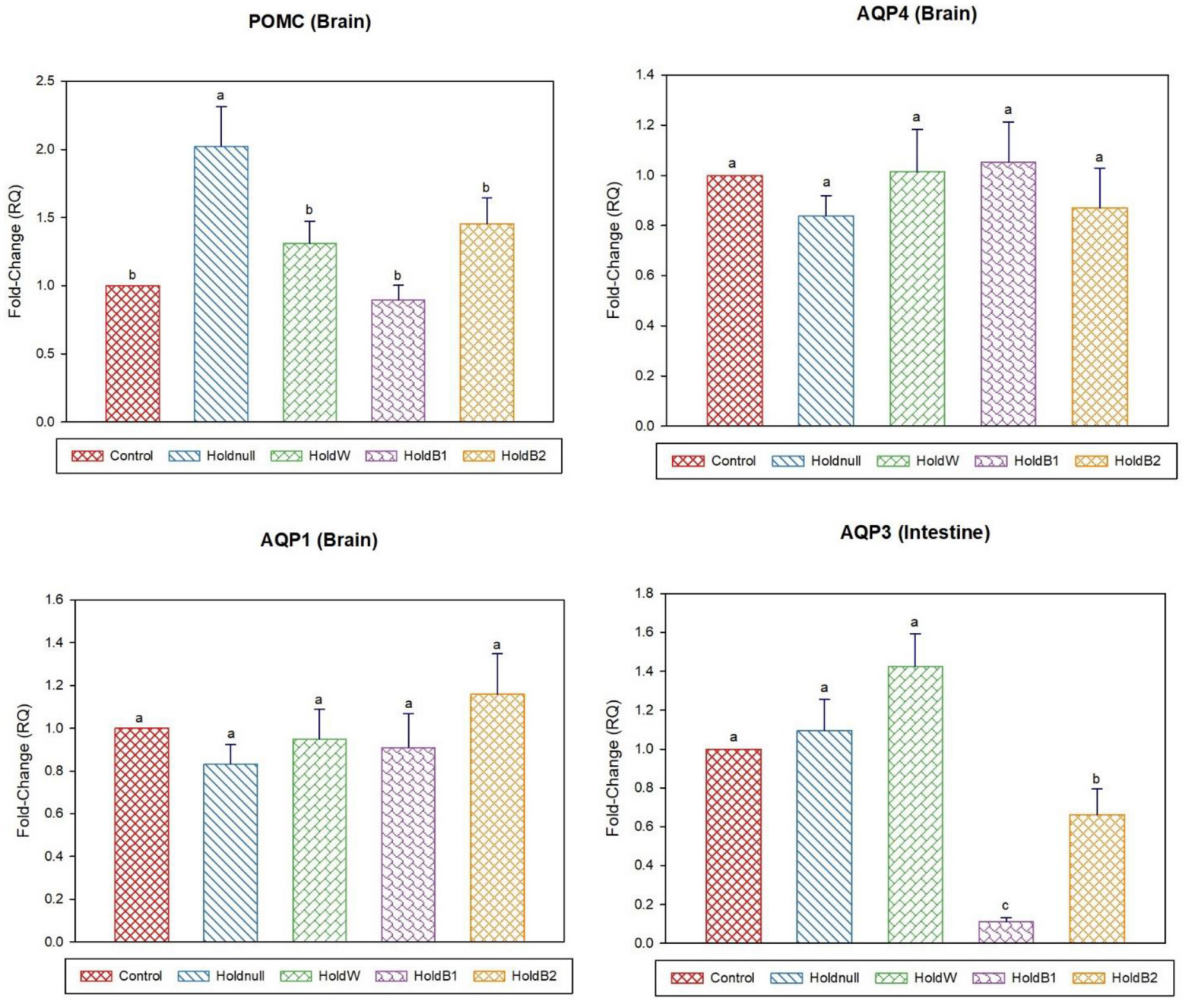
Effect of betaine on the relative expression (fold-change or RQ) of POMC, AQP4, AQP1 in the brain, and AQP3 in the intestine of day-5 old chicks. Control, placed on feed immediately; Holdnull, holding for 48 h + no access to feed or water; HoldW, holding for 48 h + water only; HoldB1, holding for 48 h + water supplemented with betaine (1ml per L); HoldB2, holding for 48 h + water supplemented with betaine (2 ml per L). Bars with different letters are significantly different (*p* < 0.05). Mean ± standard error of the mean, *n* = 6. AQP, aquaporin; POMC, proopiomelanocortin.

**Figure 4:**
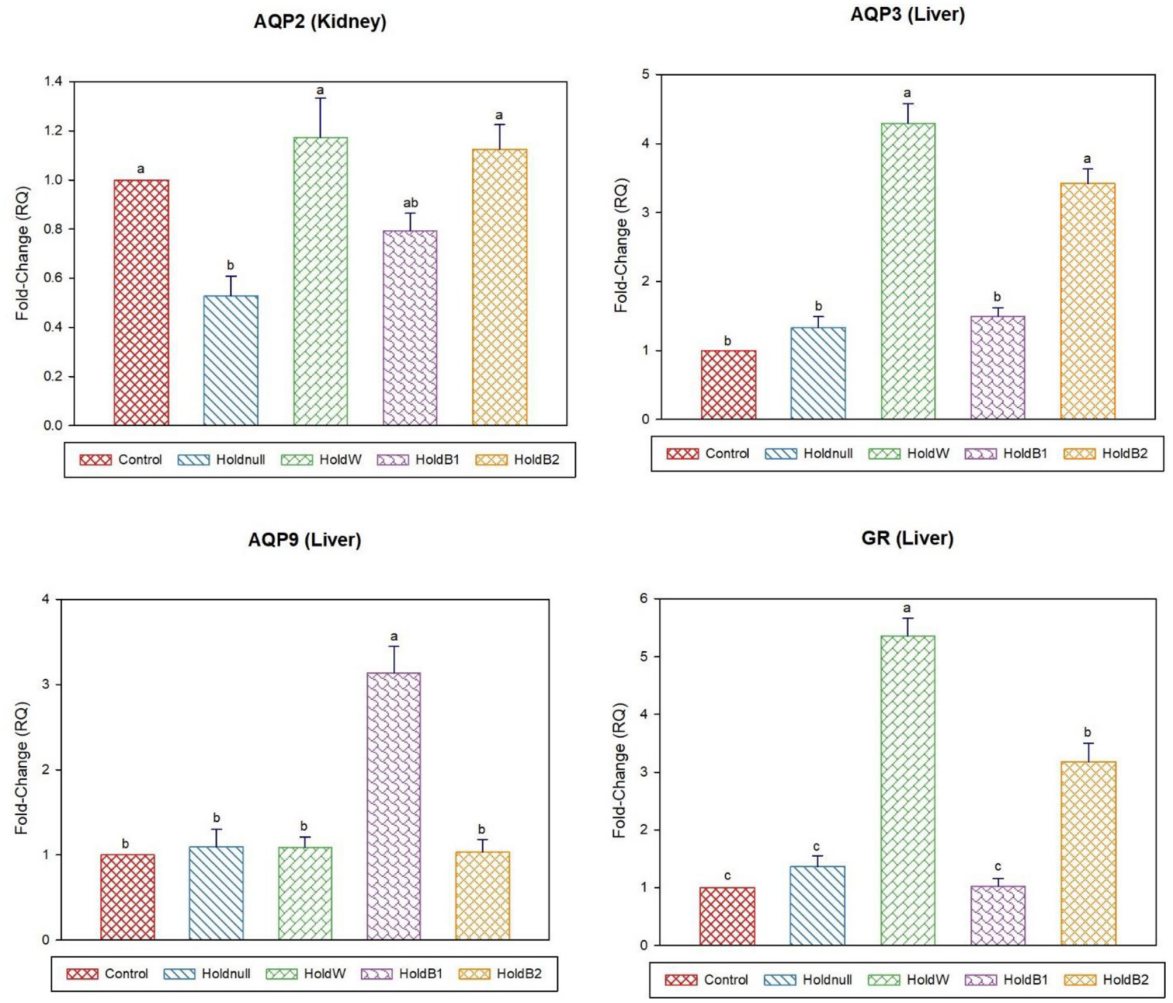
Effect of betaine on the relative expression (fold-change or RQ) of AQP3, AQP9, GR in the liver, and AQP2 in the kidney of day-5 old chicks. Control, placed on feed immediately; Holdnull, holding for 48 h + no access to feed or water; HoldW, holding for 48 h + water only; HoldB1, holding for 48 h + water supplemented with betaine (1 ml per L); HoldB2, holding for 48 h + water supplemented with betaine (2 ml per L). Bars with different letters are significantly different (*p* < 0.05). Mean ± standard error of the mean, *n* = 6. AQP, aquaporin; GR, glucocorticoids receptor.

On day-5, water holding still showed a significant increase in brain POMC than the control group, while no effects on the brain, intestine and liver aquaporins (Figures 3, 4). Renal APQ2 showed a significant decrease. Plain water supplementation restored the previous changes, while it increased hepatic AQP3 and GR. Betaine-supplemented water during holding ameliorated the impacts on stress-related transcripts (brain POMC and GR), renal AQP2, and hepatic aquaporins.

**Figure 5:**
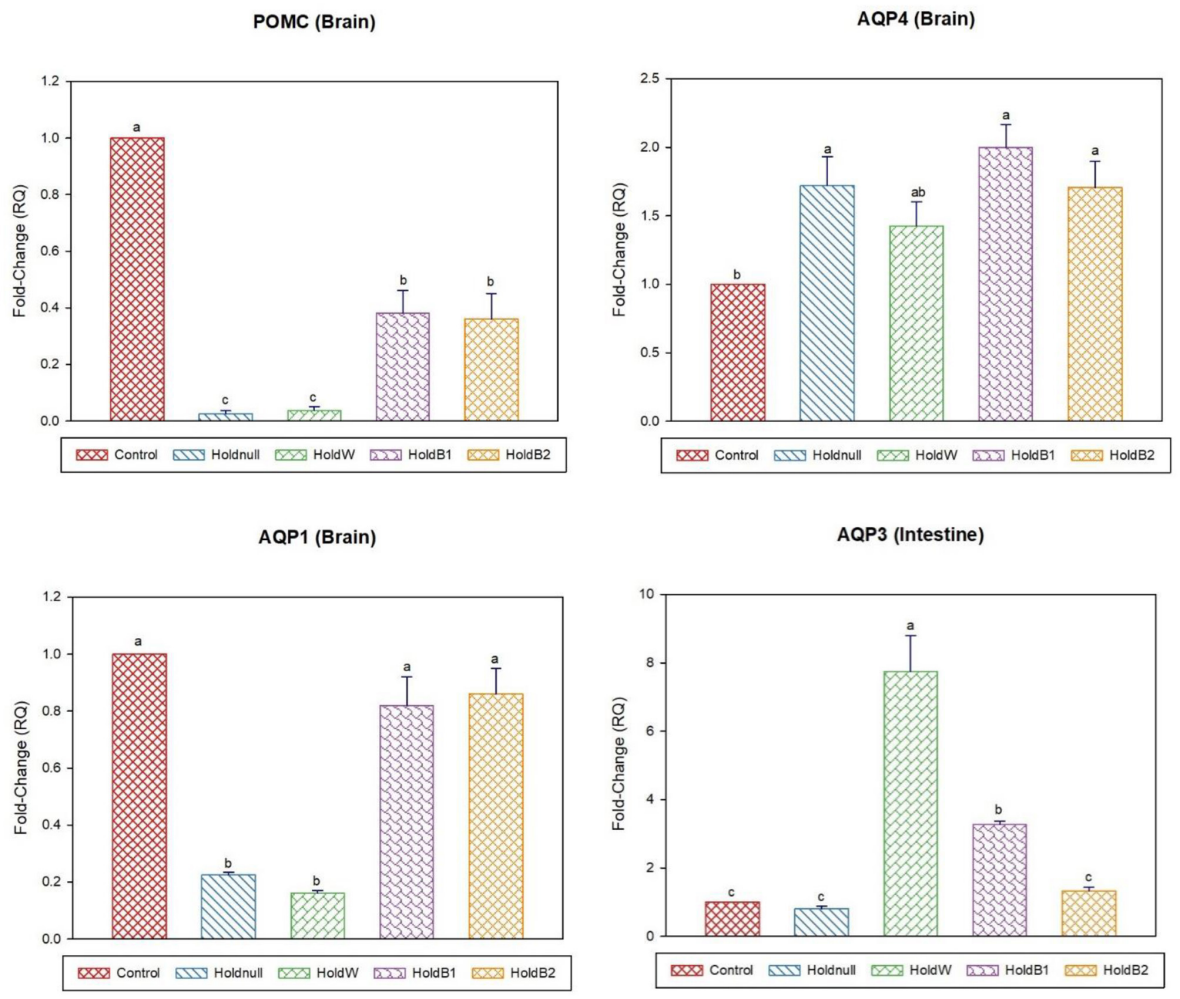
Effect of betaine on the relative expression (fold-change or RQ) of POMC, AQP4, AQP1 in the brain, and AQP3 in the intestine of day-15 old chicks. Control, placed on feed immediately; Holdnull, holding for 48 h + no access to feed or water; HoldW, holding for 48 h + water only; HoldB1, holding for 48 h + water supplemented with betaine (1 ml per L); HoldB2, holding for 48 h + water supplemented with betaine (2 ml per L). Bars with different letters are significantly different (*p* < 0.05). Mean ± standard error of the mean, *n* = 6. AQP, aquaporin; POMC, proopiomelanocortin.

**Figure 6:**
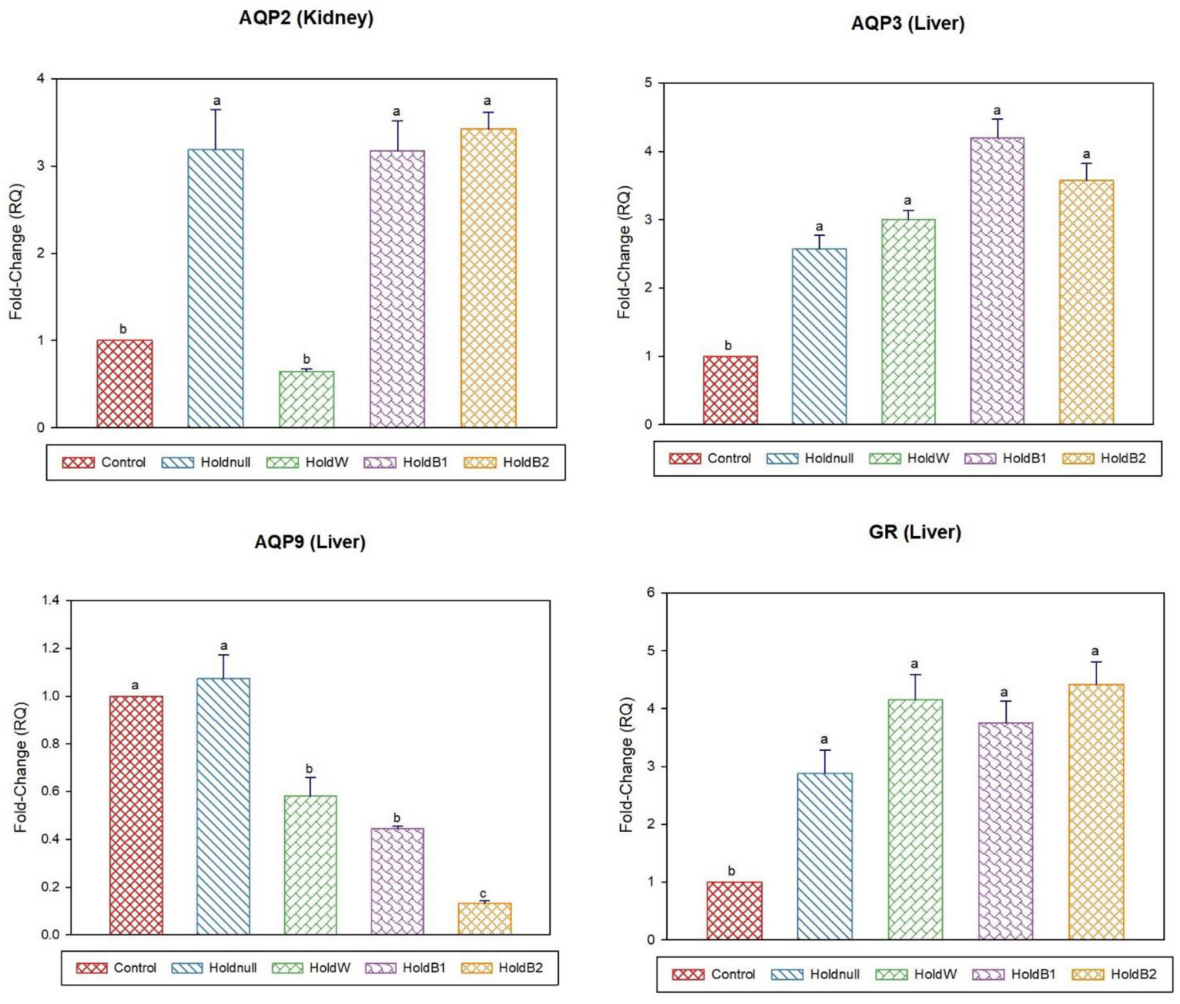
Effect of betaine on the relative expression (fold-change or RQ) of AQP3, AQP9, GR in the liver, and AQP2 in the kidney of day-15 old chicks. Control, placed on feed immediately; Holdnull, holding for 48 h + no access to feed or water; HoldW, holding for 48 h + water only; HoldB1, holding for 48 h + water supplemented with betaine (1 ml per L); HoldB2, holding for 48 h + water supplemented with betaine (2 ml per L). Bars with different letters are significantly different (*p* < 0.05). Mean ± standard error of the mean, *n* = 6. AQP, aquaporin; GR, glucocorticoids receptor. 2 AQP, aquaporin; GR, glucocorticoids receptor.

On day-15, brain aquaporins showed paradoxical expression, AQP4 increased while AQP1 decreased by water holding, while it was restored for AQP4 after water supplementation (Figures 5, 6). POMC showed a significant decrease, while renal AQP2 and hepatic AQP3 significantly increased with water holding with no effect on hepatic AQP9. Moreover, water supplementation increased intestinal AQP3 with paradoxical effects on hepatic aquaporins, AQP3 and AQP9. Betaine-supplemented water during holding maintained increased expressions of renal AQP2, hepatic AQP3, and GR, brain APQ1, AQP4, and reduced the brain POMC expression with higher expression than water holding group. Betaine-supplemented water also restored the intestinal AQP3, while it reduced hepatic AQP9.

### Correlation of Different mRNA Transcripts

On day 2, POMC showed an inverse correlation with intestinal AQP3 while positive correlation with hepatic GR, AQP3, brain APQ4, AQP1, and renal AQP2. Moreover, on the liver level, AQP9 negatively correlated with GR and AQP3. GR positively correlates with AQP3. On the brain level, all measured parameters (POMC, APQ1, and AQP4) are positively correlated to each other (Table 5).

**Table 5:** The correlation coefficient (r) between different mRNA transcripts means on day-2^1^^,^^2^.

	**Intestine AQP3**	**Liver GR**	**Liver AQP9**	**Liver AQP3**	**Brain POMC**	**Brain AQP4**	**Brain AQP1**	**Kidney AQP2**
Intestine AQP3	1.000							
Liver GR	−0.638	1.000						
	*0.247*							
Liver AQP9	−0.093	−0.580	1.000					
	*0.882*	*0.305*						
Liver AQP3	−0.677	0.994	−0.546	1.000				
	*0.209*	*0.001*	*0.341*					
Brain POMC	−0.671	0.586	−0.423	0.646	1.000			
	*0.215*	*0.300*	*0.477*	*0.239*				
Brain AQP4	−0.596	0.705	−0.671	0.718	0.893	1.000		
	*0.289*	*0.184*	*0.216*	*0.172*	*0.041*			
Brain AQP1	−0.309	0.364	−0.640	0.348	0.651	0.872	1.000	
	*0.613*	*0.547*	*0.245*	*0.566*	*0.234*	*0.054*		
Kidney AQP2	−0.869	0.933	−0.310	0.951	0.682	0.704	0.335	1.000
	*0.056*	*0.020*	*0.612*	*0.013*	*0.205*	*0.184*	*0.581*	

1 *Values are Pearson correlation coefficients and the corresponding p-values (in italic)*.

2 *AQP, aquaporin; GR, glucocorticoids receptor; POMC, proopiomelanocortin*.

On day 5, there was a negative correlation with brain POMC and hepatic AQP9. On the liver level, GR positively correlates with AQP3. On the brain level, there was a negative correlation between POMC and AQP4 (Table 6).

**Table 6:** The correlation coefficient (r) between different mRNA transcripts means on Day-5^1^^,^^2^.

	**Intestine AQP3**	**Liver GR**	**Liver AQP9**	**Liver AQP3**	**Brain POMC**	**Brain AQP4**	**Brain AQP1**	**Kidney AQP2**
Intestine AQP3	1.000							
Liver GR	0.559	1.000						
	*0.327*							
Liver AQP9	−0.823	−0.385	1.000					
	*0.087*	*0.523*						
Liver AQP3	0.376	0.971	−0.296	1.000				
	*0.533*	*0.006*	*0.629*					
Brain POMC	0.460	0.116	−0.532	0.080	1.000			
	*0.435*	*0.853*	*0.357*	*0.898*				
Brain AQP4	−0.235	0.039	0.571	−0.008	−0.887	1.000		
	*0.704*	*0.950*	*0.315*	*0.990*	*0.045*			
Brain AQP1	−0.126	0.296	−0.307	0.443	−0.250	−0.133	1.000	
	*0.840*	*0.629*	*0.615*	*0.455*	*0.686*	*0.832*		
Kidney AQP2	0.218	0.685	−0.289	0.718	−0.469	0.336	0.754	1.000
	*0.724*	*0.202*	*0.638*	*0.172*	*0.426*	*0.581*	*0.141*	

1 *Values are Pearson correlation coefficients and the corresponding p-values (in italic)*.

2 *AQP, aquaporin; GR, glucocorticoids receptor; POMC, proopiomelanocortin*.

Paradoxically, on day 15, POMC showed an inverse correlation with hepatic GR and AQP3. Additionally, on the brain level, POMC still showed a negative correlation with AQP4 while returned to the same positive correlation with AQP1 that was observed on Day-2. On the liver level, still GR positively correlates with AQP3, while AQP9 negatively correlates with AQP3 (Table 7).

**Table 7:** The correlation coefficient (r) between different mRNA transcripts means on Day-15^1^^,^^2^.

	**Intestine AQP3**	**Liver GR**	**Liver AQP9**	**Liver AQP3**	**Brain POMC**	**Brain AQP4**	**Brain AQP1**	**Kidney AQP2**
Intestine AQP3	1.000							
Liver GR	0.482	1.000						
	*0.411*							
Liver AQP9	−0.251	−0.791	1.000					
	*0.684*	*0.111*						
Liver AQP3	0.297	0.886	−0.751	1.000				
	*0.627*	*0.045*	*0.144*					
Brain POMC	−0.449	−0.731	0.163	−0.580	1.000			
	*0.449*	*0.161*	*0.794*	*0.305*				
Brain AQP4	0.000	0.704	−0.478	0.910	−0.601	1.000		
	*0.999*	*0.185*	*0.415*	*0.032*	*0.284*			
Brain AQP1	−0.532	−0.345	−0.273	−0.116	0.855	−0.155	1.000	
	*0.356*	*0.570*	*0.657*	*0.853*	*0.065*	*0.804*		
Kidney AQP2	−0.564	0.384	−0.344	0.599	−0.270	0.809	0.172	1.000
	*0.322*	*0.523*	*0.570*	*0.286*	*0.661*	*0.097*	*0.783*	

1 *Values are Pearson correlation coefficients and the corresponding p-values (in italic)*.

2 *AQP, aquaporin; GR, glucocorticoids receptor; POMC, proopiomelanocortin*.

## Discussion

The importance of early access to nutrients is well-recognized in poultry production. Previous research has indicated that delayed nutrition impaired performance and induced various physiological changes in chicks. Our results confirmed previous reports that delayed chick feeding for 48 h post-hatch resulted in reduced feed intake and weight gain during the first two weeks of life (1, 7, 35, 36). Chicks in the Holdnull group consumed 54, 27, and 19% less feed during the periods 0-5, 5-10, and 10-15 d, respectively, compared to those in the control group. Consequently, the chicks in the Holdnull group gained 57, 26, and 17% less weight compared to their counterparts in the control group at the end of these periods. The most significant impact of delayed placement on the growth performance of chicks took place during the first five days post-hatch, and then chicks started to compensate, as evidenced by the improvement in performance with advancing age. It has been reported that chicks delayed feed access early in life may recover fast and reach similar market weight as those placed immediately if the period of fasting is no longer than 24 h (37–39). As the duration of fasting increases beyond 24 h, the severity of performance loss increases (40). The reduction in performance are attributed mainly to the impairment of the gastrointestinal tract of the bird, which reduced nutrient utilization (41–44). Early access to drinking water with or without betaine did not improve performance in the current study compared to the Holdnull group. We hypothesized that betaine could elicit some beneficial effects as an osmolyte during pre-placement holding stress and, therefore, help chicks maximize body water retention and maintain cell integrity, thus improving subsequent performance. Our results regarding providing water alone agree with those reported by Noy and Sklan (10) and Fairchild et al. (1), who found that water provision during post-hatch holding time had no effect on the subsequent live performance of broiler chicks.

The yolk is the sole source of energy and protein for the newly hatch chicks prior to placement (45). Feed deprived chicks undergo dramatic changes in the physiology and metabolism to mobilize substrates for energy. In the current study, blood glucose for the feed deprived chicks was almost similar to the control group after 48 h of holding, and the chicks in the HoldB2 group had 27% higher blood glucose than the Holdnull group. Our results are in agreement with Noy and Sklan (46) and Richards et al. (47), who found no differences in blood glucose levels between chicks fasted for 48 h and early fed chicks. On the contrary, Peebles et al. (48) reported elevated blood glucose levels for fasted chicks at 48 h post-hatch. When there is no access to feed, chicks rely mainly on fatty acid oxidation as an essential energy source (49). Also, chicks convert some non-carbohydrate precursors like glycerol from triglycerides and AA to glucose through gluconeogenesis and utilize liver glycogen through glycogenolysis (50, 51). The high serum uric acid levels in the feed deprived groups 48 h post-hatch in the current study could be a sign of body protein (or yolk protein) oxidation for energy. Serum uric acid levels are reported to increase substantially during fasting in poultry (52, 53). The elevated uric acid levels in the HoldB2 during feed deprivation may be partially attributed to the oxidation of methionine for energy. Excess betaine could have transferred methyl groups to homocysteine molecules for the synthesis of methionine in the methionine cycle (54). Methionine is a glucogenic amino acid, which can be converted into glucose through gluconeogenesis. Furthermore, serum uric acid levels were positively correlated with the liver GR (*r* = 0.809) and brain POMC (*r* = 0.675) 48 h post-hatch in the current study, which could explain the association of increased level of uric acid and AA degradation occurred in the feed deprived chicks. Also, uric acid positively correlates with the liver AQP3 (*r* = 0.859), which acts in part to transport non-chargeable molecules such as ammonia that will be incorporated into uric acid (55).

In the current study, chicks corresponding to the HoldW, HoldB1, and HoldB2 had increased serum triglycerides compared to both the control and the Holdnull groups after 48 h post-hatch. Interestingly, serum triglycerides increased as a function of betaine, and this trend lasted until day 15 of age. In general, the increase in serum triglycerides in feed deprived chicks could be due to increased lipid mobilization from both the liver and yolk sac in response to energy demand. Ghasemi and Nari (56) and Yusuf et al. (57) reported that the triglycerides levels in the blood to increase as a function of betaine supplementation in chickens. The mechanism underlying the triglycerides' elevation with betaine supplementation is unclear but the correlation analysis showed that there is a direct correlation with the GR expression (*r* = 0.0894) and with hepatic AQP3 (*r* = 0.0894). We speculate that AQP3 can also transport unchangeable materials such as glycerol in the hepatic cells during fasting, which will be used in the biosynthesis of triglycerides through the action of glucocorticoid hormone on GR (58, 59). The Holdnull group had the lowest serum triglycerides level among the delayed groups, and this may emphasize the role of osmotic conditions on lipolysis. Where, hyperosmolarity was reported to reduce FFA release from rat epididymis fat pads (60).

There are different kinds of aquaporins that are expressed in various kinds of mammalian cells, among them AQP2, which is solely dependent on vasopressin release from the hypothalamus (22, 61). The current results showed variable expressions of aquaporins in different organs of day-2,−5, -and−15 old chicks with a significant correlation between different aquaporins and with the endocrine signals from the brain to reach the ultimate goal of water homeostasis. Proopiomelanocortin (POMC) is the precursor of pituitary melanocyte-stimulating hormone (α-MSH), adrenocorticotropin hormone (ACTH), and β-endorphin and considers as a marker of entire body stress condition (62, 63). On day 2, water holding significantly altered gene expression of POMC and different aquaporins; increased POMC and hepatic GR as well as selectively increased kidney AQP2, brain AQP4, and liver AQP3. POMC negatively regulated intestinal AQP3, while it showed a positive correlation with hepatic AQP3. Meanwhile, it directly correlates with brain aquaporins AQP4 and AQP1 and renal AQP2. These results indicated varied responses of different aquaporins to water restrictions. Several reports showed different behaviors of AQP expression in response to water deprivation; for example, water restriction of rats significantly increased renal AQP2 and AQP3 while no effects on AQP4 expression was observed (64). Interestingly, the expression of AQP1 showed paradoxical regulation by cortisol; levels of mRNA and protein are upregulated in the intestine but downregulated in the kidney in response to the hormone (65, 66). Our results showed that even within the same organ, the response of AQP to water restriction and stress is different that coincides with previous patterns observed by Martinez et al. (65) and MacIver et al. (66). For instance, results showed that hepatic AQP3 and AQP9 are negatively correlated throughout the experimental duration. Notably, GR sowed positive regulation of liver AQP3 throughout the experimental durations that indicates a concomitant response of water uptake by hepatocytes during water deprivation and osmotic stress. Conversely, brain AQP4 showed positive correlations with POMC in day-2 chicks while it was negatively correlated with POMC by the advancement of age, day-5 and day-15, which might indicate various sensitivity of neurons associated with osmotic stress and their maturity. Reports showed that AQP4 expression is affected by the time of astrocytes appearance in the brain (67). As expected, water supplementation relieved most of the altered expressions in POMC, GR, and AQP as a negative feedback mechanism for maintaining homeostasis (22). However, betaine showed a concomitant increase in AQP expression, particularly for brain AQP1 and AQP4, renal AQP2, and hepatic AQP3, with positive correlation with GR expression. Interestingly, AQP1 and AQP2 showed a concomitant increase in expression alongside the betaine supplementation, which indicates a positive effect of hypertonicity on AQP2 and AQP1 expression (68, 69).

In conclusion, supplemental betaine induced some changes in blood biochemical indices without improving chicks' performance during the first 15 days of life. However, betaine supplementation could ameliorate the stressful effects of water deprivation on POMC and GR expression and maintain cellular osmosis through osmolytes interactions with aquaporins expression, particularly the AQP1 and AQP2 expressions. The results also showed different behaviors of different aquaporins to osmotic stress, which requires further investigation about the molecular mechanisms underlying this regulation.

## Data Availability Statement

The original contributions presented in the study are included in the article, further inquiries can be directed to the corresponding author.

## Ethics Statement

The animal study was reviewed and approved by the Research Ethics Committee at King Saud University (Riyadh, Saudi Arabia; Ethics Reference No: KSU-SE-20-54). Written informed consent was obtained from the owners for the participation of their animals in this study.

## Author Contributions

AA-S, AA-Ab, and RA conceived and designed the experiment. AA-S, AA-Ab, EH, IS, HA, and RA performed the experiment. RA analyzed the data. RA and IS wrote the manuscript. SA-M, MQ, and AA-Az revised the paper. All authors have read and approved the final manuscript.

## Conflict of Interest

The authors declare that the research was conducted in the absence of any commercial or financial relationships that could be construed as a potential conflict of interest.
